# Field-Effect Transistor Biosensors for Biomedical Applications: Recent Advances and Future Prospects

**DOI:** 10.3390/s19194214

**Published:** 2019-09-28

**Authors:** Cao-An Vu, Wen-Yih Chen

**Affiliations:** Department of Chemical and Materials Engineering, National Central University, Taoyuan City 32001, Taiwan

**Keywords:** FET biosensor, biomedical application, nanotransducer, field-effect transistor, aptamer, antibody

## Abstract

During recent years, field-effect transistor biosensors (Bio-FET) for biomedical applications have experienced a robust development with evolutions in FET characteristics as well as modification of bio-receptor structures. This review initially provides contemplation on this progress by analyzing and summarizing remarkable studies on two aforementioned aspects. The former includes fabricating unprecedented nanostructures and employing novel materials for FET transducers whereas the latter primarily synthesizes compact molecules as bio-probes (antibody fragments and aptamers). Afterwards, a future perspective on research of FET-biosensors is also predicted depending on current situations as well as its great demand in clinical trials of disease diagnosis. From these points of view, FET-biosensors with infinite advantages are expected to continuously advance as one of the most promising tools for biomedical applications.

## 1. Introduction:

Since being introduced by Clark in 1962 [1], biosensors have been widely employed in diverse applications such as cancer diagnosis [2], toxicity detection [3], food analysis [4], health prognosis [5], etc. Biosensors, analytical devices converting biological responses into electrical signals [6], usually consist of at least two basic components combining together: A biological receptor and a physical-chemical transducer [7]. The former converts responses from the biochemical domain, usually an analyte concentration, into a chemical or physical output signal with a defined sensitivity whereas the latter transfers the signal from the output domain of the bio-recognition system, mostly to the electrical domain [7]. Among various kinds of biosensors, field-effect transistor biosensors (Bio-FETs), an integrated between bio-receptors and ion-sensitive field-effect transistors (ISFET), emerged as the most developed candidates because of several advantages. Indeed, roughly a half of century since the invention of initial ISFET generation by Bergveld in 1970 we have witnessed the evolutions of these transducers in numerous biosensor applications [8]. In a typical FET system, the sensing elements are immobilized on the sensing channels (semiconductor path), which are connected to source (S) and drain (D) electrodes, to capture the targets (usually via high specificity and binding affinity). A bias potential is applied and modulated to a third electrode (gate). The channel conductance, which is varied by detection of the targets, is recorded and further processed by an electrical measurement system. There are two kinds of FETs: n-type with electrons as the main charge carriers and p-type with holes as the primary charge carriers. In an n-type FET system, if the probes detect positively charged molecules, the charge carriers (electrons) will accumulate on the sensing channels and increase the conductance. If negatively charged targets are recognized, the conductance will be decreased due to the depletion of the electrons. Conversely, for a p-type FET system, binding with positive charges results in conductance decline due to a reduction of the charge carriers (holes) and capturing negative charges raises the conductance because of hole accumulation. Outburst of nanotechnology triggers combination between biosensors and nanomaterials for sensing application with breakthrough designs in which biomolecules (antibodies, nucleotides and so on) as receptors are immobilized on the surface of nanotransducers (nanowires, nanotubes, nanoparticles, etc.).

Materials for the transducers are one of the most important components that need to be considered to fabricate FET biosensors. The two most popular nanomaterials attracting particular interests of scientists are silicon-based (silicon nanowire—SiNW) and carbon-based (graphene and carbon nanotubes—CNTs) ones. Silicon nanowire has been demonstrated not only high-sensitivity for label-free and real-time detection but also potentially commercialized because of feasibility in mass-production from the semiconductor industry [9,10,11,12,13,14]. However, in spite of continuous advancement in large-scale assembly techniques (fluid flow directed assembly [15], bubble-blown assembly [16], contact assembly [17], electric-field-assisted assembly [18], Langmuir-Blodgett assembly [19], field-assisted electrospinning [20], the “place then grow” method [21] and superlattice nanowire pattern transfer [22]) with a well-controlled dimension, orientation and density of synthesized nanowires [23], high device-to-device variation and low carrier mobility are primary issues crucially overcome on the way to commercialize SiNWFETs [24]. Since SiNWFET-based sensors have been successfully employed to detect a variety of biological and chemical molecules (proteins [9,10,11,12,13,25], nucleic acids [14,25,26,27,28], viruses [29,30] and different targeted substances [9,31,32,33,34]), there have been plentiful strategies to improve their sensing capability by operating detection in subthreshold regime [35], using frequency domain measurement [36], optimizing surface modification with electrical field alignment [37], integrating with nanopore morphology [38] or electrokinetic devices [39], and fabricating branched nanowires [40] for clinical diagnosis, the ultimate goal of profuse biomedical applications [41]. CNTs, whereas, possess superior physical and chemical properties for biosensor applications such as: High mechanical strength, surface area and aspect ratio, excellent chemical and thermal stability [42]; outstanding conductivity for nanoscale transducers [43,44,45] and ideal semiconducting behavior as nanoscale FETs [46]; especially advantageous to immobilize bio-probes (antibodies, enzymes, oligonucleotides, etc.) either in their hollow cavity or on nano-surfaces [47,48,49,50,51] and efficient in promoting electron-transfer reactions [52]. Nevertheless, synthesizing CNTs always produces a mixture of semiconducting (-s) and metallic (-m) tubes, which degrades electrical performance and creates device-to-device variation, imposing a huge disadvantage for CNTs as FET applications [24]. Similar to SiNW, since CNTs has been demonstrated as one of materials for FET [53,54], two primary tactics have been developed to subdue aforementioned difficulty and assemble CNT arrays: Direct- and post-synthesized separation of s-/m- CNT mixtures [55]. Nonetheless, integrating CNTs into large-scale devices has still been challenging partially due to their low current output, small active areas and heterogeneous s-/m- mixtures [24]. Graphene, a two-dimensional layer with honeycomb lattice formed by covalent σ bonds between carbon atoms [56], has outstanding mechanical strength (known as the strongest material with 130 GPa tensile strength and 1000 GPa modulus) [57], ultra-large specific area (~2630 m^2^/g) [58], especially extraordinary electrical properties with high mobility of charge carriers (more than 2 × 10^5^ cm^2^ V^−1^ s^−1^) [59], which is very advantage for ultra-fast charge transport and therefore raise particular interest on graphene as materials for FET applications. However, a lack of band gap leading to small on/off ratio in graphene FETs is its disadvantage in this field [24]. Graphene can be synthesized by top-down (exfoliation) and bottom-up (chemical vapor deposition—CVD) processes. The oldest exfoliating method to obtain single graphene sheets comprises of expanding graphitic layers by oxidization, producing single layers of graphene oxide by exfoliation, removing oxygen groups by reduction [24]. Nevertheless, no reduction method (chemical, thermal and electrochemical treatments) can completely remove oxygen functional groups, which (within defects from oxidation step) severely degrades electrical performances of reduced GO compared to pristine graphene [56,60]. The drawbacks of other exfoliating methods are unable to collect large graphene sheets by mechanical exfoliation and diversified flakes formed by various amount of layers with electrical characteristics deviated by chemicals used in sonication exfoliation [24]. Similar to top-down process, mechanical and electrical properties of graphene grown by CVD are also affected due to defects and impurities caused during this fabrication procedure [24]. Therefore, absence of mass-production of perfectly structured graphene is another hindrance for commercialization of graphene as material of semiconducting industry [56].

Biosensing element is the other crucial factor that needs to be taken into account in designing FET biosensors. In the dawn of these FET-based devices, antibodies and DNAs/RNAs are prevailing candidates mainly because they are simple, low-cost to synthesize, can be immobilized on a wide range of various surfaces and provide fast detection via high specificity and affinity binding with the target molecules, which are usually protein and nucleic acids in most of biomedical applications. Nevertheless, on one hand, not only is synthesizing antibodies from animals expensive and inappropriately commercialized but also coverage of immobilization areas is far less than full due to stearic hindrances from bulky structure of antibodies [61]. On the other hand, DNA and RNA probes require high ionic strength conditions to shield their intermolecular repulsive force for hybridizations [24]. Both of them weaken or mislead detected signal by FETs in physiological environments with high screening effect (also known as Debye length). Besides, guanine–cytosine-rich sequences on nucleic acid structures, which form stem-loop structures as well as self- and cross-dimers, also contribute to false detection results from nonspecific binding [62]. The subsequent periods thus have witnessed an exploration of new generations including antibody fragments (Fab, Fab’ and scFv), aptamers, peptide nucleic acids (PNA), locked nucleic acids (LNA) and neutralized DNA (nDNA) as bio-probes in order to subjugate these drawbacks.

During the most recent years, efforts primarily concentrate on improving sensitivity of FET devices via breakthrough of nanotransducers and/or probe design. The former approach includes inventing novel fabrication methods as well as discovering new materials for nanotransducers whereas the latter strategy focuses on continuing exploiting compact-structured recognition elements (aptamer and antibody fragments) within controlling immobilized orientation and optimizing probe density as well as incredible strategies of signal enhancements in order to overcome limitations of screening effect in high ionic strength of physiological environments. This review centralizes on notable advances in FET research and development of FET biosensors for biomedical applications from 2016 to early 2019, dividing into three categories: Evolutions of nanotransducers, antibody and its fragments as bio-probes, and nucleic acid as bio-probes. References are limited to published articles and the others reported before this period are also exploited as additional resources in order to provide the fundamental knowledge in this field as well as depict the explosion of FET-biosensors before this period.

## 2. Evolution of Nanotransducers for FET-Based Sensors

Graphene is one of conventional materials and have been over-exploited for FET-biosensors since being invented. Though, spotlight on improvements of graphene FET (GFET) as nanotransducers has been continuous thus far. Researchers from China invented a variant of GFET biosensors in which graphene foam was used as electrical channel to detect adenosine triphosphate (ATP). An extremely large surface area and ultra-high sensitivity possessed by porous/hollow structures of three-dimensional (3D) graphene foam produced an ATP biosensor with a limit of detection (LOD) of 0.5 pM in a wide linear range from 0.5 pM to 50 μM, which is several orders lower than previous reports and thus opens a door for trace sensing of ATP at the picomolar level in a biological system [63]. Another modification on the GFET structure comes from a research group in America who embedded an Al_2_O_3_ layer (3 nm thickness) onto a reduced graphene oxide (rGO) channel by the atomic layer deposition (ALD) technique to prevent nonspecific binding of water or unwanted species onto the rGO surface. This layer therefore raises reproducibility and stability of the sensor without affecting its FET properties [64].

**Figure 1:**
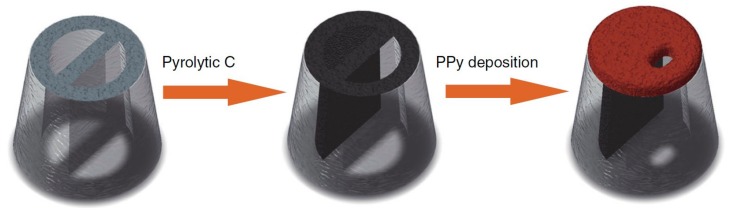
Fabrication of nanopore-extended field-effect transistor (nexFET) in dual-barrel quartz nanopipettes by depositing pyrolytic carbon in one of the barrels before electrodeposition of polypyrrole (PPy) at the carbon-coated nanopipette tip [65].

Ren’s group invented nanopore-extended FET (nexFET), which enables higher molecular throughput, enhanced signal-to-noise and heightened selectivity via functionalization with an embedded receptor. This new class of nanosensor combines advantages of nanopore platform and FETs by using a novel nanopipette-based polypyrrole (PPy) ionic FET. It is possible to tune nanopore dimensions in real time to the size of targeted molecules. Molecular transport can also be efficiently controlled at the single-molecule level by controlling gate voltage (Figure 1). Moreover, the PPy gate layer is appropriate to immobilize the artificial sensing elements for selective molecular detection (3 kpb double-stranded DNA or anti-insulin by insulin) [65].

**Figure 2:**
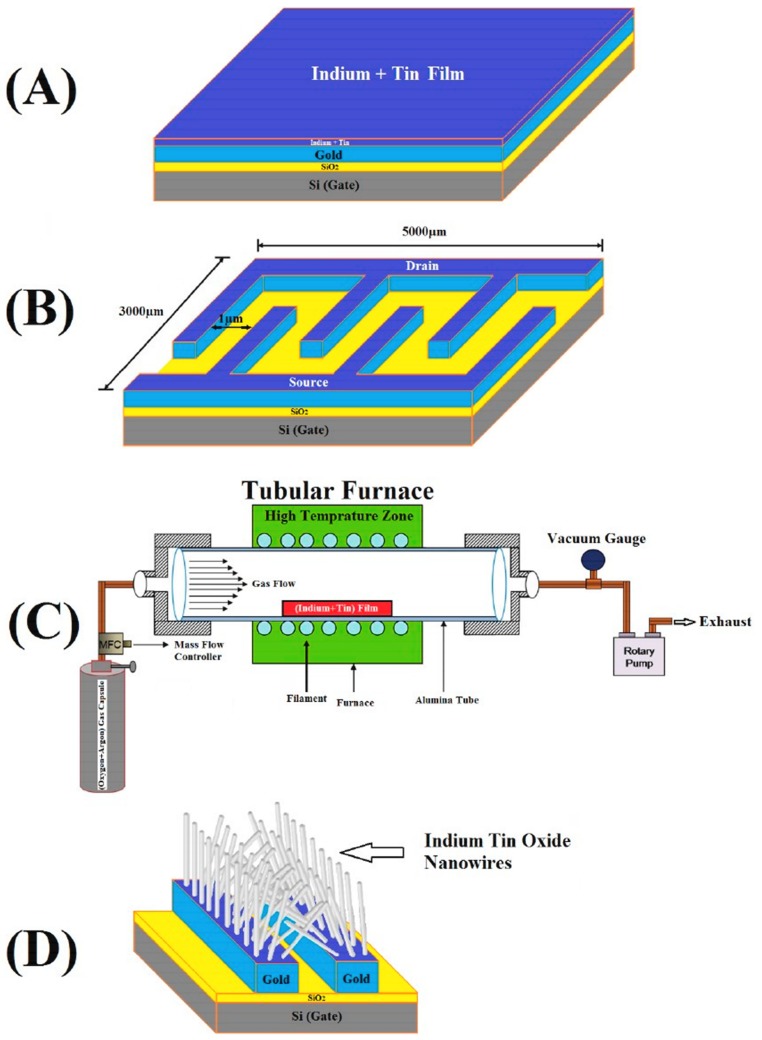
Indium-tin oxide nanowires (ITO-NW) FETs were fabricated by (**A**) coating indium and tin film onto gold film and (**B**) defining pattern of the devices by E-beam lithography before (**C**) treating them with controlled parameters in tubular furnace to (**D**) form ITO nanowires [66]. Reprinted from Biosensors and Bioelectronics, 105, Shariati, The Field Effect Transistor DNA Biosensor Based on ITO Nanowires in Label-Free Hepatitis B Virus Detecting Compatible with CMOS Technology, 58–64, Copyright 2018, with permission from Elsevier.

In order to improve probe immobilization and target hybridization, Mohsen Shariati invented a nucleic-FET sensor with indium-tin oxide nanowires (ITO NWs) as a nanotransducer. A precursor substrate for ITO nanowires was firstly prepared by depositing a 2 µm gold layer onto the SiO_2_ substrate as electrodes before sputtering an indium–tin–indium thin film onto this Au layer with 4 µm thickness of 90% indium and 10% tin. ITO nanowires was then grown in a tubular furnace at 800 °C within presence of Ar–O_2_ gases (ratio Ar:O_2_ = 20:1) in 90 minutes (Figure 2). Inheriting intensive conductance of ITO nanowires and functional modified surface after immobilizing thiolated DNA probes, their devices were capable of detecting Hepatitis B Virus (HBV) in a linear concentration range of 1 fM–10 μM (LOD ≈ 1 fM) as well as excellently discriminated among non-complementary, mismatch and complementary DNA oligonucleotide sequences. Additionally, their HBV-ITO-FET biosensors can retain stability and repeatability up to 98% and 96% initial measured signal after 3 and 5 weeks, respectively [66].

**Figure 3:**
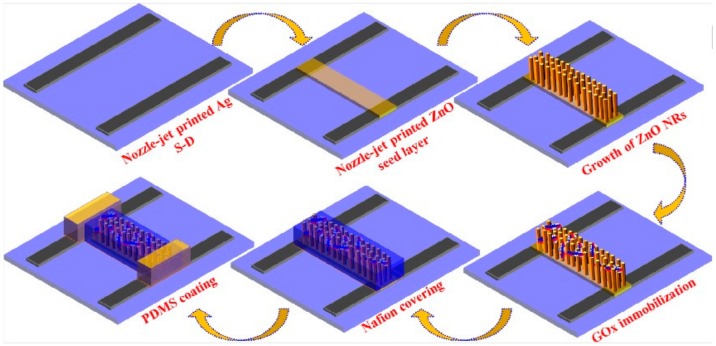
Fabrication process of ZnO-FET biosensors by the nozzle-jet printing method [67]. Reprinted from Journal of Colloid and Interface Science, 506, Bhat et al., Nozzle-Jet Printed Flexible Field-Effect Transistor Biosensor for High Performance Glucose Detection, 188–196, Copyright 2017, with permission from Elsevier Elsevier.

**Figure 4:**
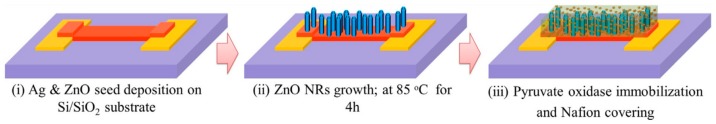
Fabrication process of ZnO-FET biosensors by the radio-frequency magnetron-sputtering method [69]. Reprinted from Journal of Colloid and Interface Science, 498, Ahmad et al., ZnO Nanorods Array Based Field-Effect Transistor Biosensor for Phosphate Detection, 292–297, Copyright 2017, with permission from Elsevier.

Despite of unpopularity in FET biosensors, zinc oxide (ZnO) with large surface area, good conductivity, and biocompatibility properties was independently exploited by two research groups of Hahn and Haghini to fabricate ZnO nanoribbon (NR) FETs (ZnO-NR-FETs) for detecting different molecules. The former nozzle-jet printed ZnO seed layers for ZnO-NRs to grow onto the polyethylene terephthalate (PET) substrate from ZnO quantum dot (ZnO-QD) ink and reached an LOD of 70 μM in glucose detection (Figure 3) [67]. The latter employed a thin layer of Al-doped ZnO (AZO) as a seed layer for ZnO-NRs growing on Au-coated glass substrate and advanced to a further achievement of 3.8 μM LOD within a linear dynamic range from 10 μM to 5 mM [68]. Hahn and his colleagues also unveiled another technique for ZnO-FETs by radio-frequency (RF) magnetron-sputtering ZnO seed layer on the Si/SiO_2_ wafer (Figure 4). They applied them for phosphate detection and harvested an impressive sensing performance (LOD = 0.5 μM in a linear range of 0.1 μM–7000 mM) compared to recently published phosphate biosensors. In their ZnO-NR-FET enzymatic sensors, pyruvate oxidase (PyO) was immobilized to detect phosphate and Nafion was used on the surface to prevent enzyme leakage and enhance sensitivity [69].

Similarly, different research groups from China and Hong Kong utilized a high surface area characteristic of the NR structure for their ISFETs. In comparison with the conventional nanowire structure, larger surface-to-volume ratio of NR increases efficient surface area for detection where the sensing elements are immobilized and therefore proportionally improves sensitivity of the sensors [70,71,72]. Another modified structure of NW called nanonets (nanostructured networks), in which NWs form a network with to randomly orientation, possess not only high surface area but also tolerance to defects to enhance reproductivity in comparison with conventional nanowires. It was therefore integrated into a p-type transistor and applied to detect DNA for the first time in 2017 [73].

**Figure 5:**
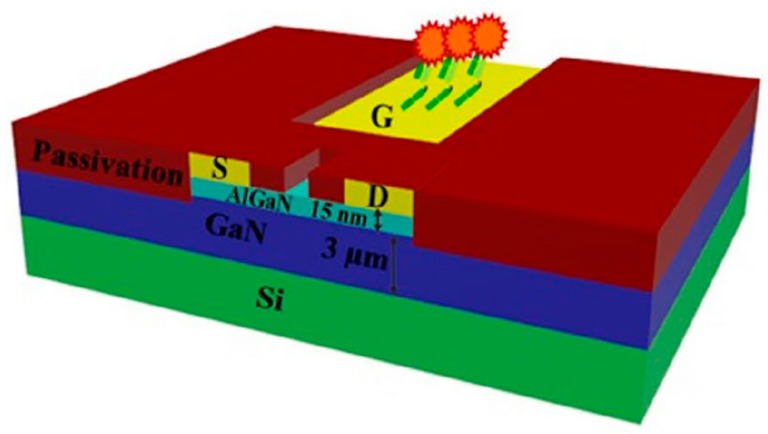
Schematic model of the AlGaN/GaN high electron mobility transistor (HEMT) with the active channel and gate electrode, which is functionalized with receptors, are passivated separately. Only these two components of this FET biosensor are exposed to the analytes [74].

In addition to silicon, GaN is among traditional materials with numerous renovated debut FET. Collaboration of various research groups from the National Tsing Hua University, Nation Central University and National Cheng Kung University designed a neoteric device to overcome detrimental effect of Debye length by using AlGaN/GaN high electron mobility transistors (HEMTs), which is chemically inert and thermally stable, for their electric-double-layer (EDL) FET biosensors. In their EDL FETs, bio-receptors are immobilized on active channel, which is separated from gate electrode (Figure 5). In comparison with conventional FET biosensors, their EDL AlGaN/GaN HEMTs not only directly detect protein in the physiological environment (1 × phosphate-buffered saline (PBS), human serum (HS)) without the diluting or washing process but also is capable of sensing uncharged molecules due to their methodology mechanism. The other advantages including simplified fabrication fast detection process, excellent sensitivity and repeatability, adjustable signal magnitude and stable baseline. Moreover, these miniaturized EDL AlGaN/GaN HEMT chips are able to be integrated with a microfluidic system and/or packaged into a polymer substrate connected with a measurement system containing a micro SD card reader and an electrical device interfaced with laptop displaying the test results. They are therefore portable and potentially applicable for personal healthcare in the future [74,75,76,77]. Employing AlGaN/GaN as materials for ISFET, Stock et al. also integrated a monolithic Wheatston bridge layout and coated a 2 nm Al_2_O_3_ layer on the gate area to yield a denser and more homogeneous probe layer for improved sensitivity and stability of the penicilinase-modified FET (WPenFET) sensors up to 60 days in comparison with ones fabricated by chemically wet oxidized method as well as preventing substantial loss of immobilized enzyme for acetylcholinesterase-modified FET (WAcFET) over 12 days [78].

In the same way in which only modifying the surface without robustly intervening the transducers, Jang et al. combined a polymer membrane onto the FET gate to surpass the Debye length limitation and detect the analyte in the physiological condition. Their sensing membrane was fabricated by mixing bio-receptor and 1-Ethyl-3-(3-dimethylaminopropyl)carbodiimide/N-hydroxysuccinimide (EDC/NHS) with poly(styrene-co-methacrylic acid) (PSMA) before spin-coating the modified membrane onto remote gate (PET/ITO substrate). The immobilized receptors in the polymer permitted binding of targeted molecules closer to the surface and depress screening effect. Consequently, their antibody-embedded polymer FET biosensors successfully detected cortisol in 1 × PBS with high sensitivity from 10 fg/mL to 10 ng/mL and LOD of 1 pg/mL [79]. Lately, Jeong et al. went beyond this border with LOD of 1 aM in the linear range of 100 aM–10 nM cortisol concentration, which was attained from abundant density of immobilized antibody due to high surface area of the transducers they developed by fabricating needle-like N-doped carbon nanofibers via electrospinning, vapor deposition polymerization (VDP) and carbonization [80].

**Figure 6:**
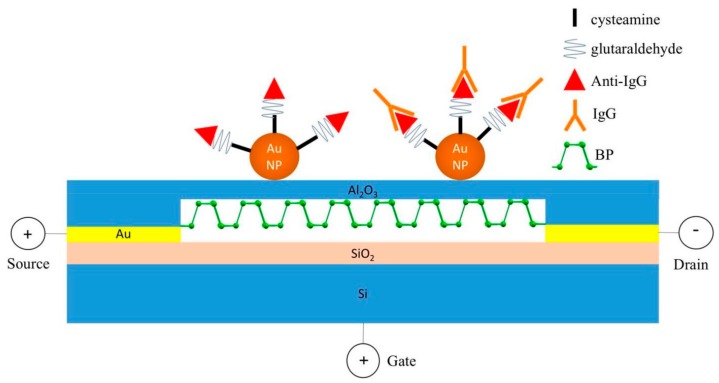
Fabrication of black phosphorus (BP)-FET biosensors with passivation of the Al_2_O_3_ dielectric layer on the surface of exfoliated BP nanosheets to prevent them from being oxidized prior to surface modification of Au nanoparticles and probe immobilization of Anti-Immunoglobulin G (Anti-IgG) [81]. Reprinted from Biosensors and Bioelectronics, 89, Chen et al., Field-Effect Transistor Biosensors with Two-Dimensional Black Phosphorus Nanosheets, 505–510, Copyright 2017, with permission from Elsevier.

New materials for nano-FET in recent years witnessed emergence of black phosphorus (BP), which is not only the most stable allotrope in the phosphorus family with an orthorhombic lattice but also is an attractive material for various electronic/optoelectronic applications because of its wide band gap range and high carrier mobility. In comparison with semi-metallic graphene and other indirect band gap metal dichalchonenides, BP possesses a direct and thickness-dependent band gap varying from 0.3 eV (bulk BP) to 2.0 eV (mono layer). In comparison with MoS_2_, BP can reach much larger field-effect extracted carrier mobility, up to ~1000 cm^2^V^−1^s^−1^ for a 10-nm thick BP nanoflake at room temperature (MoS_2_: ~200 cm^2^V^−1^s^−1^). These advantages were successfully utilized to passivate a few layers of BP nanosheets (NSs) onto Si/SiO_2_ surface as sensing channels for FET immunosensors (Figure 6) [81].

Organic materials for semiconductors have also slightly advanced. Poly-3-hexyl-thiophene (P3HT), an organic material that can provide a good level of surface coverage and uniformity for protein adsorption, was coated on the Au substrate for electrolyte-gated organic FET (EGO-FET) immunosensor of procalcitonin (PCT). Physical adsorption of the anti-PCT antibody on the P3HT-coated substrate produced the first EGO-FET, which can detect this sepsis marker with a LOD of 2.2 pM [82]. One of the most efficient organic FET (OFET) sensors was developed based on biomolecule guanine and most commonly used small molecule semiconductor pentacene for a non-biomedical application. Their combination to form layer-by-layer of guanine and pentacene on Si/SiO_2_ substrate produced a high-sensitive OFET for NO_2_ gas [83].

## 3. Antibody and its Fragments as Bio-Probes for FET Immunosensors

Antibody and its fragments are the most popular biological entities employed as bio-receptors because they can specifically bind with targeted molecules (antigen) in a familiar reaction so-called immunoassay to produce electrical signal readable by tranducers. For the first time, the Ebola glycoprotein (EGB) was detected in PBS, human serum and plasma by graphene FET. The reduced graphene surface was initially deposited with Al_2_O_3_ and Au NPs for probe immobilization. Human anti-EBOV glycoprotein antibody was then conjugated with Au NPs via surface modification of cystamine and glutaraldehyde. The fabricated rGO-FET immunosensors could detect the Ebola glycoprotein (EGP) with concentration at 1 ng/mL within a few seconds in 100-time diluted PBS, human serum and plasma, which is an ultra-low LOD and extremely fast processing time in comparison with other techniques, proving superiority of FET in this race [84]. Immunoassays of human apolipoprotein A1 (hAPOA1), a biomarker for bladder cancer, by silicon nanowire also exhibits an improved sensitivity compared to the bead-based enzyme-linked immunosorbent assay (ELISA) method with a LOD approximately 100 pg/mL [85].

Admittedly, gold nanoparticles (GNPs) is a promising candidate for surface modification to raise FET sensitivity due to its high-surface-to-volume ratio, high surface energy, facilitated electron transfer between bio-specific layer and electrode surface by conductivity. After sputter-coating rGO substrate with a thin Al_2_O_3_ layer and GNPs to boost performance, anti-*Escherichia coli* (anti-*E. coli*) was functionalized to onto these NP by glutathiol (GSH), EDC-NHS and bicinchoninic acid (BCA) linkers to detect *E. coli* with detection range of 10^3^–10^5^ CFU/mL [64]. Similarly, fabrication with Al_2_O_3_ and GNPs was included in surface modification of BPNS-FET immunosensors for the same function. In both aforementioned sensors, Al_2_O_3_ plays an important role to guarantee their reproducibility and stability without impacting the electrical properties by prevent nonspecific binding of water or unwanted species onto the sensors’ surface. Afterwards, functionalization between antibody and GNPs by cystamine and glutaraldehyde produced a human IgG BP-FET biosensor with LOD down to 10 ng/mL (Figure 6) [81]. Research groups from Russia slightly optimized surface modification on GNPs using less linking chemicals and oriented half-fragment antibody to detect prostate specific antigen (PSA) in 0.01 × PBS with an impressive dynamic range from 23 fg/mL to 500 ng/mL and 23 fg/mL (≈ 0.7 fM) LOD. Half-fragments of anti-PSA were separated by cleaving disulfide bond in heavy chain of their structures with 2-Mercaptoethylamine (MEA) and conjugated with Au NPs before being anchored onto GOPS-SH-modified silicon substrate (3-glycidopropyltriethoxysilane with thiol groups) [86,87]. Scientists in China advanced to a further step with analogous immunoassays by NR-FET in high ionic strength solution (100 mM PBS) and human plasma reached the lowest detection range of 10 pM–1 µM and 100 pM–1 µM for the first time, respectively. In comparison with conventional nanowire, increased surface area of the NR structure allows more biomolecules immobilized on the sensing channel and thus elevates sensitivity of the FET biosensors. In this study, NR-ISFET immunosensors were fabricated by surface modification of 3-(trimethoxysilyl)propyl aldehyde and mPEG-silane on the silicon NR surface prior to immobilizing the anti-PSA antibody. PEG also plays an important role to expand the sensing region under the Debye length of the physiological environment for detectable FET signals [71]. The carcinoembryonic antigen (CEA) is another cancer biomarker also detected by NR-FET with slightly improved linear detection range down to 0.01–10 ng/mL with LOD = 0.01 ng/mL in comparison with one used graphene-FET. However, these results were obtained in 0.01 × PBS solution and the whole anti-CEA antibody as bio-receptors [70]. The breakdown in this type of immunoassay, therefore, came from EDL FET immunosensors with AlGaN/GaN HEMT as a transducer and oriented probes as a sensing element. Immobilized half-fragments of anti-CEA as oriented probes on gold electrode of AlGaN/GaN HEMT through a thiol group lets their binding site towards the upside to detect CEA in 1× PBS containing 1% bovine serum albumin (BSA). Detection range could significantly decrease to 100 fM–1 nM. This result was also obtained when operating this EDL AlGaN/GaN HEMT FET sensor for immunoassays of N-terminal pro b-type natriuretic peptide (NT-proBNP; whole antibody as bio-probe) in similar condition. Furthermore, EDL AlGaN/GaN HEMT FET immunosensors of NT-proBNP was also applicable in serum sample and achieved good linearity with sample concentrations of 180.9 pg/mL, 269.2 pg/mL, 660.8 pg/mL, 1848 pg/mL, 3008 pg/mL, 4596 pg/mL and 5000 pg/mL [74,75,77].

**Figure 7:**
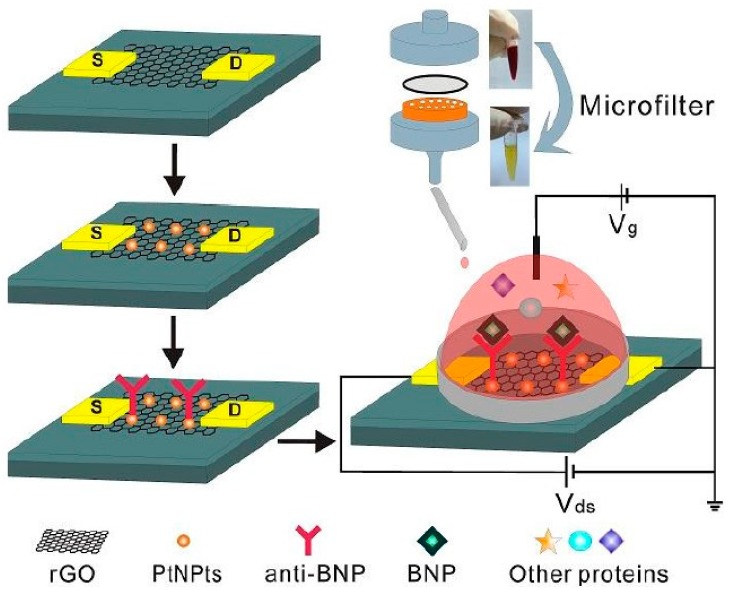
Integration of a custom-made microfilter to platinum nanoparticles (PtNPs)-decorated reduced graphene oxide (rGO) FET biosensors [88]. Reprinted from Biosensors and Bioelectronics, 91, Lei et al., Detection of Heart Failure-Related Biomarker in Whole Blood with Graphene Field Effect Transistor Biosensor, 1–7, Copyright 2017, with permission from Elsevier.

In late 2016, Zhang and colleagues became the pioneer group successfully integrating a sample pretreatment system into GFET biosensors to detect BNP in whole blood. In their report, they deposited platinum nanoparticles (PtNPs) onto the GFET surface in order to increase electrical conductivity before immobilizing anti-BNP by EDC/NHS surface modification. The PtNP-decorated GFET immunosensors were then integrated with a custom-made microfilter and 400 nm pore polycarbonate membranes for blood-cell removal before delivering to fabricated GFET channels for BNP sensing, which also achieved an LOD at 100 fM in 0.001 × PBS and BNP concentration down to 50 nM in whole blood (Figure 7) [88]. Two years later, a research group from Germany surpassed this border with a rGO-ISFET device, which can detect the lowest concentration of NT-proBNP from 1 to 10 (pg/mL) with calculated LOD of 30 pg/mL, which is under the threshold value stage 1 of heart failure (100 pg/mL for men and 125 pg/mL for women according to the New York Heart Association) and permits diagnosis of health conditions [89].

Human immunodeficiency virus (HIV) is another global public health concern associated with cardiovascular disease (CVD) and rheumatoid arthritis (RA). Some recent months ago, Indian and Korean scientists from various research groups developed an ultrasensitive smart nanosensor based on graphene that can detect biomarkers of three aforementioned diseased within a linear range of 1 fg/mL–1 μg/mL as well as LOD of 100 fg/mL (p24—HIV biomarker) and 10 fg/mL (cardiac Troponin 1 (cTn1)—CVD biomarker and cyclic citrullinated peptide (CCP)—RA biomarker). All of them are literately the lowest LOD in comparison with achievements of previous publications, establishing a new milestone in sensing technology of these biomarkers [90]. Label-free detection of lung cancer and liver cancer also witnessed a further step with multianalyte of cytokeratin fragment 21-1 (CYFRA 21-1, biomarker for lung cancer) and α-fetoprotein (AFP, biomarker for liver cancer) was simultaneously detected in human serum. Their quantitative analysis achieved LOD of 1 ng/mL (CYFRA 21-1) and 10 ng/mL (AFP) in the linear range of 1–100 ng/mL, opening a future for clinical diagnosis of multiple biomarkers in human serum [91].

Diagnosing pesticide is another biomedical application where FET also recently imprinted and immediately impressed with its prestige by successfully analyzing chlorpyrifos, a pesticide inhibiting acetylcholinesterase and responsible for severe neurological, autoimmune and persistent developmental disorders in humans with long-term treatment. A biosensor developed from microfluidic-based graphene FET and anti-chlorpyrifos antibodies can detect this antigen in spiked sample at LOD down to 1.8 fM in the linear range from 1 fM to 1 μM, outstripping LOD accomplished by previous techniques and setting up a landmark in sensing technology of this biomolecule [92].

Additionally, antibody also functions in non-biomedical applications, for instance, to detect the plum box virus (PPV)—a pathogen responsible for an infectious disease on a stone fruit tree named Sharka. In combination between electronic-gated organic FET (EGOFET), anti-PPV IgG and protein G (for highly uniform and oriented antibodies immobilized on the sensing surface), EGOFET-biosensors were capable of operating in a dynamic range from 5 ng/mL to 50 μg/mL with LOD of 180 pg/mL PPV concentration [93].

## 4. Nucleic Acid Probes for FET Biosensors

Nucleic acid-based sequence is another bio-recognition factor famous for specific binding with targeted ligands. Nucleic acid-based molecule is one of the two most popular kinds of biomarkers that FET sensors with a DNA/RNA probe can detect. During recent years, there have been efforts to improve sensitivity of FET nucleic acid sensors at all cost.

For the first time, integrating organic-charge modulated FETs (OCMFETs) with hairpin-shaped probes not only enhances performance but also opens the future for a new class of low-cost, easy-operation and portable genetic sensors (Napoli’s group) [94]. OCMFET is an ideal candidate for performing detection measurements in the aqueous environment because of its physical separation between sensing area and organic semiconductor. In comparison with other feasible conformations, hairpin-probe has been reported with excellent results in different applications, especially for DNA sensors operated by on-off mechanism in label-based mode. The sensor was prepared by surface modification with hairpin-shaped DNA as recognition element via thiol group and 6-mercapto-1-hexanol (MCH) as a spacer and blocking agent. The fabricated sensor can detect DNA hybridization with target concentration as low as 100 pM [94].

Manufacturing FET biosensors for small DNA detection, Gao and co-scientists advanced to a further breakthrough of an approximately 20,000 × improvement in sensitivity (compared to regular sensitivity in the nM range of a 20-mer FET-DNA sensor) by an engineered hairpin DNA probe allowing target recycling and hybridization chain reaction. Their proposed strategy was applicable for 21-mer DNA detection at sub fM targeted concentration with high specificity against single-base mismatch sequences. Similar to Napoli’s group, a hairpin DNA was immobilized onto GFET surface via 1-pyrenebutyric acid N-hydroxysuccinimide ester (PBASE) in N,N-dimethylformamide surface modification. The novelty of this technique comes from DNA helpers, which can trigger target recycling and hybridization chain reaction, lead to signal enhancement of biorecognition events and therefore rocket sensitivity up to 20,000 × (LOD ≈ 5 fM for 21-mer detection) compared to previous publications [95]. Another exclusive design of nucleotide-probe comes from neutralized DNA (nDNA; developed from a research activated in 2013 [27]), in which two partially neutralized DNA exhibited better sensitivity and selectivity than the regular and fully neutralized DNA in hybridization (reached LOD of 0.1 fM) [28]. nDNA can also greatly discriminate between perfect- and mismatched sequences of GC-rich single nucleotide polymorphisms (GC content: 75%) although the hybridization should be carried out in low ionic strength environment (10 mM Bis-Tris propane) in order to maximize the discrimination effect [62].

Improvements of low cost MoS_2_-FET DNA sensors have continuously appeared in this period, kicked-off with phosphorodiamidate morpholino oligos (PMO), a third generation of antisense oligonucleotides, as a recognition element. In comparison with DNA, PMO has a neutral backbone of morpholine rings, which weakly affects PMO-DNA hybridization behavior and therefore deteriorates noise impact onto sensitivity and detection signal. Incorporation PMO with MoS_2_-FET for the first time not only increased detectability of DNA sensor with LOD down to 6 fM in 0.5 × PBS but also produced a DNA sensor with potential usability for real-sample detection after being proved to detect 10 fM and 1 pM target in 10 × diluted human serum. However, there still have been some limitations remained, especially fabrication of MoS_2_ nanosheet onto SiO_2_/Si substrate (drop-casting) as well as uncontrolled orientation of surface modification and hybridization process [96]. Following the trend, a research group in Beijing overcome aforementioned drawback of surface modification by optimizing their probe density with AuNP functionalization before immobilizing a DNA sequence to capture its complementary target, a biomarker of down syndrome. Their MoS_2_-based DNA sensors are capable of detecting this biomarker with LOD below 100 aM and respond with its concentration as low as 1 fM in real-time operation. The fabricated sensors also exhibited outstanding specificity and selectivity with response up to 240% in comparison with previously reported MoS_2_ FET biosensors, totally appropriate for down syndrome screening [97]. Move onto this race, laboratories from Portugal achieved a lowest LOD of single nucleotide polymorphism (SNP) discrimination at 25 aM with their graphene FET (GFET) sensors [98].

A NR-ISFET operating in the dual-gate mode could detect 20-mer DNA of *Cordyceps sinensis* (CorS), one of the rarest golden worms and most precious traditional Chinese medicines (TCM) with multi-medical functions, with LOD as low as 50 pM. A 20-mer DNA probe was settled down on NR-ISFET surface via APTES and glutaraldehyde to detect three types of 20-mer target including its complementary (20-mer DNA of CorS), 5-mer mismatch and fully noncomplementary sequences. On one hand, in comparison with conventional ISFET, their NR-ISFET biosensors possibly operated with smaller sample amount and shorter detection time. On the other hand, in comparison with single-gate mode, sensitivity and specificity were remarkably elevated [72].

It is also possible to immobilize nucleic acid probes on FET channel for protein detection. Among the nucleic acid probes for protein detection, aptamer emerged as an elite candidate because it is capable of binding with a variety protein at high affinity and specificity. Especially, aptamers are promising recognition factors for replacing bulky antibody in immunoassays by FET because their compact size mostly under the Debye length and binding events with bio-species are hence attracted closer to the sensing surface for potential changed easily to be recorded. General sensitivity and selectivity of FET-immunosensors is therefore improved. Indeed, aptamers were anchored onto the EDL AlGaN/GaN HEMT FET transducers mentioned in Section 2 to detect the C-reactive protein (CRP) and Human Immunodeficiency Virus-1 Reverse Transcriptase (HIV-1 RT) in 1 × PBS containing 1% BSA and in human sera. The former was sensed from 1 fM to 100 nM (covering from low to high risk regions of cardiovascular disease), especially in the rank of 9–26 nM (regarded as middle risk of cardiovascular disease) and the latter could be recognized with concentration from 1 fM to 10 pM [74,76]. Employing aptamer to recognize HIV-1, scientists from Malaysia, however, also only reached an LOD at 600 pM with a multiwall carbon nanotube FET as a transducer [99]. An important leap in HIV-1 assays by aptamers came from a novel liquid-ion gated FET biosensors of Iran researchers, which can detect HIV DNA in a linear range of 1 aM–10 nM and achieve 0.3 aM LOD. Moreover, their FET biosensors are potentially applicable for clinical applications because of good sensitivity and high recovery in real blood samples [100].

Singh et al. developed and extended-gate FET with inter-digitated gold microelectrodes (IDµE) to detect malaria biomarker plasmodium falciparum glutamate dehydrogenase (PfGDH) in serum samples by a 90-mer ssDNA (thiolated aptamer) selective to PfGDH. In comparison with similar research, their reported sensor exhibited a comparable LOD with 16.7 pM PfGDH in spiked buffer and 48.6 pM PfGDH in serum samples as well as a superior detection range of 0.1 pM–10 nM for both types of analytes [101]. Hao et al. modified GFET substrate with PBASE to immobilize insulin-specific aptamer IGA3 and was able to detect insulin in Krebs–Ringer bicarbonate (KRB) buffer with a concentration ranging from 100 pM to 1 µM and LOD of 35 pM [102].

In addition to compact structure and high affinity with specific proteins, another advantage of aptamers is capability of conformational change after binding with their corresponding targets. Together with obstructions from the Debye screening length, detection of low- or un-charged ligands unremarkably affect transconductance of the FET and result in indistinguishable binding events through the electrical signal. Conformational changes in charged receptors of aptamers, which happened within or in proximity of the Debye length, can generate enough variation of surface potential to overcome this issue. Nakatsuka and his teammates exploit this function for sensing small molecules (serotonin, dopamine, glucose and sphingosine-1-phosphate (S1P)) by FETs in physiological solutions. In this study, aptamers with stem-loop structures were selected as bio-receptors for their FET biosensors. Out-put signal modulation triggered by conformational change of negatively phosphodiester backbones after detecting insignificant-charged targets are recognizable and processable by electrical measurement systems [103].

## 5. Conclusions and Future Prospects

As described from the beginning, this review concentrates on FET-related developments in biomedical applications during the most recent periods. To this end, we have initially flashed back to the early history of ISFET with its invention and operating mechanism before quickly reviewing the transducers and bio-receptors, the two most essential constituents of FET biosensors. This rapid introduction provides a general background to assist the readers in following the focal point with remarkable studies of this field during the 2016–2019 timeline analyzed in subsequent sections and summarized in Table 1.

Applying FET-nanosensors in clinical trials to detect biomolecules is not only severely hindered by ionic screening effect caused by high ionic strength of physiological environment (salt concentration is higher than 100 mM), in terms of electrical signal detection, but also is critically obstructed by the presence of multiple proteins and ligands in serum samples, in terms of sensing capability. However, while there have been numerous publications proposing solutions for the former, the effort to overcome detriments of the latter is still very limited. Blocking unbinding surface after the immobilization process is the most popular strategy with ethanolamine, BSA, 6-mercapto-1-hexanol (MCH) as usual blocking agents. Majority of the solution for the Debye screening length focused on performing detection in diluted analytes with low ionic strength and/or employing small molecules as bio-receptors (antibody fragments, DNA aptamer and RNA aptamer). Congo red is the latest compact recognition factor since it was employed as a replacement for antibody and feasibly measure Aβ fibrils in human serum with concentration ranging from 100 pM to 10 μM [104]. Hideshima and his colleagues revealed a totally different approach to this obstacle by proposing sodium dodecyl sulfate (SDS), an ionic surfactant, for signal enhancement of sensing buckwheat protein (BWp16), an allergen in processed food. Their surfactant-induced signal amplification method produced additional negative charges, coming from SDS coupling with BWp16, enough for conductance change detected by FET after binding with ethanolamine-capped anti-BWp16 Fab fragments [108].

**Figure 8:**
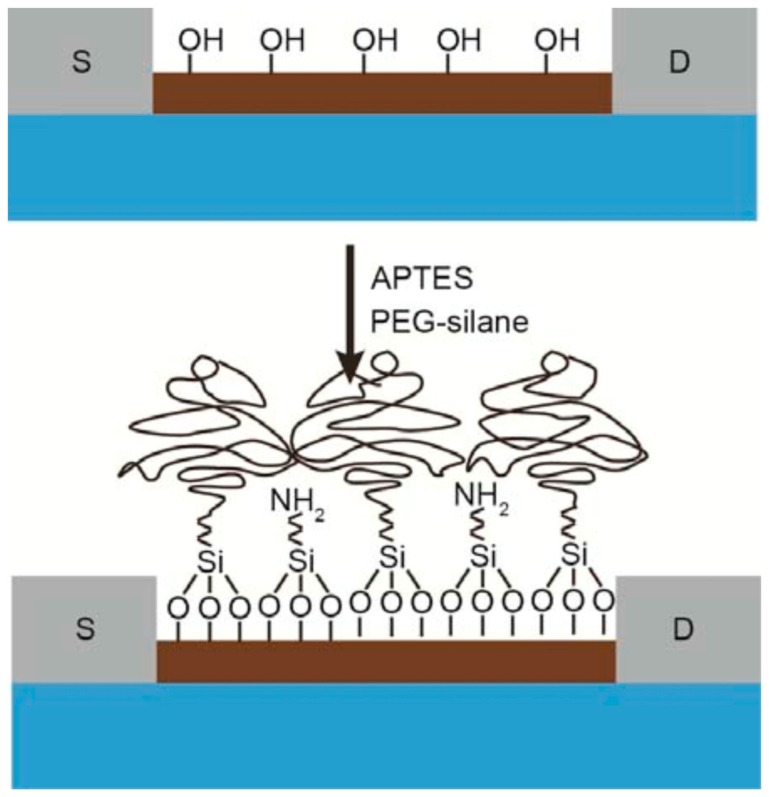
Surface modification of silicon nanowire surface with mixed-SAMs constituting of APTES and PEG-silane [109]. Reprinted with permission from Nano Letters, 15, Gao et al., General Strategy for Biodetection in High Ionic Strength Solutions Using Transistor-Based Nanoelectronic Sensors, 2143–2148. Copyright 2015 American Chemical Society.

**Figure 9:**
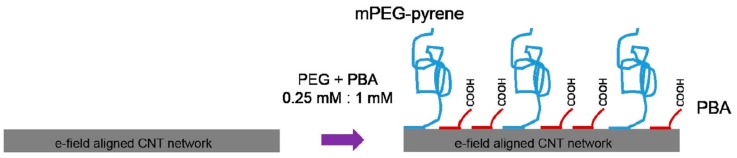
Surface modification of the CNT network surface with mixed-SAMs constituting of PBA and mPEG-pyrene [107]. Reprinted from Sensors and Actuators B: Chemical, 255, Filipiak et al., Highly Sensitive, Selective and Label-Free Protein Detection in Physiological Solutions Using Carbon Nanotube Transistors with Nanobody Receptors, 1507–1516, Copyright 2018, with permission from Elsevier.

Gao et al. kicked-off a trend of mixed self-assembled monolayers (mixed-SAMs) to overcome this hindrance by modifying the SiNW surface with polyethylene glycol (PEG) to expand detectable region for biosensing prostate specific antigen (PSA) in 150 mM phosphate buffer (PB; Figure 8) [109]. Tarasov’s group advanced to a further step by introducing a series that proposed mixed-SAMs as an exclusive surface modification tactic combining with various kinds of antibody fragments [105,106,107,110]. Two of them exploited 10 kDa mPEG and F(ab’)_2_ receptors to detect thyroid-stimulating hormone (TSH) in whole serum with LOD below 500 fM (by extended-gated metal-oxide semiconductor FET (MOSFET) with gold sensing channel), which has five orders of magnitude lower LOD and significant prevention of nonspecific binding [105], and 10 fM (by electrolyte-gated GFET) [106]. Furthermore, a detailed study about this topic to elucidate working mechanism of PEG on ionic screening of analyte charges has recently been completed by means of simulation [110]. The other employing camelid heavy-chain VHH antibody fragments (VHH: camelid single domain antibodies, also called nanobodies), one of the shortest biological receptors (molecular weight ≈ 13 kDa and length <3 nm). Components of mixed-SAMs are 1-pyrenebutyric acid (PBA) and mPEG-pyrene (molecular weight ≈ 10 kDa; Figure 9) [107]. Their fabricated CNT-FET immunosensors could detect green fluorescent protein (GFP) from under 1 pM to 10 nM. However, instead of carrying an anti-fouling function, extending the sensible area under the Debye length is the primary role of mPEG-pyrene in their series [105,106,110], which allows them to obtain roughly three-fold signal enhancement [106]. Especially, in comparison with similar research using antibody fragments, theirs are the only sensors that can implement immunoassays in 100 mM and whole serum whereas sensing environments of the others are 10 mM or more diluted buffer [105,106,107,110]. Shifting to surface modification with mixed-SAMs therefore become a potentially dual-functional strategy to subdue detriments of high ionic strength and unwanted binding caused by multiple proteins in serum environments. These mixed-SAMs are feasibly constituted from a linker and an anti-fouling factors in which PEG-derived and zwitterionic materials are promising candidates to partially prevent interfered proteins from approaching the sensor surface as well as obstructing specific binding of probes and targets via formation of hydrated layers. Moreover, it is necessary to consider the blocking surface, which avoids non-specific binding between un-modified linkers and targets, as a crucial step to improve anti-interference functions of FET biosensors. Ultimately, antibody fragments and aptamers with their compacts structure and well-controlled orientations are irresistible to optimize sensitivity of FET-biosensors. In summary, future FET biosensors are potentially combinations of all aforementioned parts (compact and oriented probes, linkers with anti-fouling functions and blocking agents) for biomedical applications, especially serving in clinical sensing and medical diagnosis.

## Figures and Tables

**Table 1:** Summary of remarkable publications of FET biosensors for biomedical applications from 2016 to early 2019.

Transducer Material	Bioprobe	Target Molecule	Analyte	LOD	Linear Range	Ref.
Graphene foam	ATP Aptamer	ATP	0.1 × HS	0.5 pM	0.5 pM–50 µM	[63]
Reduced Graphene Oxide	Anti-*E. coli*	*E. coli*	Deionized water River water	10^3^ CFU/mL 10^4^ CFU/mL	10^3^–10^5^ CFU/mL	[64]
Indium Tin Oxide Nanowire	DNA	HBV DNA	100 mM PBS	1 fM	1 fM–10 µM	[66]
Zinc Oxide Nanoribbon	GOx	Glucose	100 mM PBS	70 µM	0.0–80 mM	[67]
Zinc Oxide Nanoribbon	GOx	Glucose	10 mM PBS	3.8 µM	10 µM–5 mM	[68]
Zinc Oxide Nanoribbon	PyO	Phosphate	0.02 M HEPES	0.5 µM	0.1 µM–7 mM	[69]
Silicon Nanoribbon	Anti-CEA	CEA	0.01 × PBS	0.01 ng/mL	0.01–100 ng/mL	[70]
Silicon Nanoribbon	Anti-PSA	PSA	100 mM PBS HS	10 pM 100 pM	10 pM–1 µM 100 pM–1 µM	[71]
Silicon Nanoribbon	DNA	DNA of CorS	0.1 × PBS	50 pM	50 pM–1 μM	[72]
Aluminum Gallium Nitride	HIV-1 Aptamer Half anti-CEA CRP Aptamer CRP Aptamer Anti-NT-proBNP	HIV-1 RT CEA CRP CRP NT-proBNP	1 × PBS 1 × PBS 1 × PBS HS HS		1 fM–10 pM 100 fM–1 nM 1 fM–100 nM 0.34–23.2 mg/L 180.9 pg/mL– 5 ng/mL	[74]
Indium Tin Oxide	Anti-Cortisol	Cortisol	1 × PBS	1 pg/mL	10 fg/mL–10 ng/mL	[79]
Needle-like Carbon Nanofiber	Anti-Cortisol	Cortisol	PBS	100 aM	100 aM–10 nM	[80]
Black Phosphorus	Anti-IgG	Human IgG	0.01 × PBS	10 ng/mL	10–500 ng/mL	[81]
Poly-3-hexyl-thiophene	Anti-PCT	PCT	PBS	2.2 pM	0.8 pM–4.7 nM	[82]
Graphene	Human anti-EGP	EGP	0.01 × PBS, HS, Human Plasma	1 ng/mL	1–444 ng/mL	[84]
Silicon Nanowire	Anti-APOA1	hAPOA1	0.01 × PBS	1 ng/mL	100 pg/mL–10 μg/mL	[85]
Silicon Nanoribbon	Anti-PSA	PSA	0.01 × PBS	23 fg/mL	23 fg/mL–500 ng/mL	[86]
Reduced Graphene Oxide	Anti-BNP	BNP	0.001 × PBS	100 fM	100 fM–1 nM	[88]
Reduced Graphene Oxide	Anti-NT-proBNP	NT-proBNP	HS	10 pg/mL		[89]
Graphene	Anti-p24 Anti-cTn1 Anti-CCP	p24-HIV cTn1 CCP	50 mM PBS 50 mM PBS 50 mM PBS	100 fg/mL 10 fg/mL 10 fg/mL	1 fg/mL–1 μg/mL 1 fg/mL–1 μg/mL 1 fg/mL–1 μg/mL	[90]
Silicon	Anti-AFP Anti-CYFRA 21-1	AFP CYFRA 21-1	HS HS	10 ng/mL 1 ng/mL	1–100 ng/mL 1–100 ng/mL	[91]
Graphene	Anti-Chlorpyrifos	Chlorpyrifos	Standard PB	1.8 fM	1 fM–1 μM	[92]
Pentacene	Anti-PPV	PPV	50 mM PBS	180 pg/mL	5 ng/mL–50 μg/mL	[93]
Organic	Hairpin-shaped DNA	DNA	TE 1 M NaCl	100 pM	100 pM–10 nM	[94]
Graphene	Hairpin-shaped DNA	DNA	5 × saline-sodium citrate	5 fM		[95]
Molybdenum Disulfide	Phosphorodiamidate Morpholino Oligos	DNA	0.5 × PBS 10 × diluted HS	6 fM	10 fM–1 nM 10 fM–1 pM	[96]
Molybdenum Disulfide	DNA	DNA	0.1 × PBS	100 aM	100 aM–1 fM	[97]
Graphene	DNA	DNA	10 mM PB, 150 mM NaCl, 50 mM MgCl_2_	25 aM	1 aM–100 fM	[98]
Multiwall Carbon Nanotubes	HIV-1 Aptamer	HIV-1 Tat	Blood sample	600 pM	0.2 nM–1 µM	[99]
Nickel Oxide	DNA	HIV DNA	0.01 M PBS	0.3 aM	1 aM–10 nM	[100]
Silicon	PfGDH Aptamer	PfGDH	HS 50 mM K_3_PO_4_, 50 mM NaCl, 5 mM KCl, 2.5 mM MgCl_2_	48.6 pM 16.7 pM	100 fM–10 nM 100 fM–10 nM	[101]
Graphene	Insulin Aptamer	Insulin	PBS	35 pM	100 pM–1 µM	[102]
Indium (III) Oxide	Dopamine Aptamer Serotonin Aptamer S1P Aptamer Glucose Aptamer Glucose Aptamer	Dopamine Serotonin Lipid S1P Glucose Glucose	1 × PBS, 1 × aCSF 1 × PBS, 1 × aCSF 1 × HEPES 1 × Ringer buffer Whole blood diluted with 1 × Ringer buffer		10^−14^–10^−9^ M 10^−14^–10^−9^ M 10 pM–100 nM 10 pM–10 nM 10 μM–1 mM	[103]
Silicon	Congo Red	Aβ fibrils	HS		100 pM–10 μM	[104]
Metal Oxide	F(ab’)_2_ of anti-TSH	TSH	Serum	500 fM	500 fM–10 nM	[105]
Graphene	F(ab’)_2_ of anti-TSH	TSH	Serum	10 fM	0.8 fM–1 nM	[106]
Singlewall Carbon Nanotubes	Nanobody	GFP	100 mM Tris		<1 pM–10 nM	[107]

## References

[1] Clark L.C., Lyons C. (1962). Electrode Systems for Continuous Monitoring in Cardiovascular Surgery. Ann. N. Y. Acad. Sci..

[2] Tothill I.E. (2009). Biosensors for Cancer Markers Diagnosis. Semin. Cell Dev. Biol..

[3] Ligler F.S., Taitt C.R., Shriver-Lake L.C., Sapsford K.E., Shubin Y., Golden J.P. (2003). Array Biosensor for Detection of Toxins. Anal. Bioanal. Chem..

[4] Bunney J., Williamson S., Atkin D., Jeanneret M., Cozzolino D., Chapman J., Power A., Chandra S. (2017). The Use of Electrochemical Biosensors in Food Analysis. Curr. Res. Nutr. Food. Sci..

[5] Pantelopoulos A., Bourbakis N.G. (2010). A Survey on Wearable Sensor-Based Systems for Health Monitoring and Prognosis. IEEE Trans. Syst. Man Cybern. Syst..

[6] Chaplin M.F., Bucke C. (1990). Biosensors. Enzyme Technology.

[7] Thevenot D.R., Toth K., Durst R.A., Wilson G.S. (2001). Electrochemical Biosensors: Recommended Definitions and Classification. Biosens. Bioelectron..

[8] Bergveld P. (1970). Development of an Ion-Sensitive Solid-State Device for Neurophysiological Measurements. IEEE Trans. Biomed. Eng..

[9] Cui Y., Wei Q., Park H., Lieber C.M. (2001). Nanowire Nanosensors for Highly Sensitive and Selective Detection of Biological and Chemical Species. Science.

[10] Zheng G., Patolsky F., Cui Y., Wang W.U., Lieber C.M. (2005). Multiplexed Electrical Detection of Cancer Markers with Nanowire Sensor Arrays. Nat. Biotechnol..

[11] Patolsky F., Zheng G., Lieber C.M. (2006). Fabrication of Silicon Nanowire Devices for Ultrasensitive, Label-Free, Real-Time Detection of Biological and Chemical Species. Nat. Protoc..

[12] Stern E., Klemic J.F., Routenberg D.A., Wyrembak P.N., Turner-Evans D.B., Hamilton A.D., LaVan D.A., Fahmy T.M., Reed M.A. (2007). Label-Free Immunodetection with CMOS-Compatible Semiconducting Nanowires. Nature.

[13] Huang Y.-W., Wu C.-S., Chuang C.-K., Pang S.-T., Pan T.-M., Yang Y.-S., Ko F.-H. (2013). Real-Time and Label-Free Detection of the Prostate-Specific Antigen in Human Serum by a Polycrystalline Silicon Nanowire Field-Effect Transistor Biosensor. Anal. Chem..

[14] Lin C.-H., Hung C.-H., Hsiao C.-Y., Lin H.-C., Ko F.-H., Yang Y.-S. (2009). Poly-Silicon Nanowire Field-Effect Transistor for Ultrasensitive and Label-Free Detection of Pathogenic Avian Influenza DNA. Biosens. Bioelectron..

[15] Huang Y., Duan X., Wei Q., Lieber C.M. (2001). Directed Assembly of One-Dimensional Nanostructures into Functional Networks. Science.

[16] Yu G., Cao A., Lieber C.M. (2007). Large-Area Blown Bubble Films of Aligned Nanowires and Carbon Nanotubes. Nat. Nanotechnol..

[17] Fan Z., Ho J.C., Jacobson Z.A., Yerushalmi R., Alley R.L., Razavi H., Javey A. (2008). Wafer-Scale Assembly of Highly Ordered Semiconductor Nanowire Arrays by Contact Printing. Nano Lett..

[18] Duan X., Huang Y., Cui Y., Wang J., Lieber C.M. (2001). Indium Phosphide Nanowires as Building Blocks for Nanoscale Electronic and Optoelectronic Devices. Nature.

[19] Acharya S., Panda A.B., Belman N., Efrima S., Golan Y. (2006). A Semiconductor-Nanowire Assembly of Ultrahigh Junction Density by the Langmuir–Blodgett Technique. Adv. Mater..

[20] Zheng Z., Gan L., Zhai T. (2016). Electrospun Nanowire Arrays for Electronics and Optoelectronics. Sci. China Mater..

[21] Weiss N.O., Duan X. (2013). A Guide for Nanowire Growth. Proc. Natl. Acad. Sci. USA.

[22] Heath J.R. (2008). Superlattice Nanowire Pattern Transfer (SNAP). Acc. Chem. Res..

[23] Jia C., Lin Z., Huang Y., Duan X. (2019). Nanowire Electronics: From Nanoscale to Macroscale. Chem. Rev..

[24] Zhang A., Lieber C.M. (2016). Nano-Bioelectronics. Chem. Rev..

[25] Cheng S., Hotani K., Hideshima S., Kuroiwa S., Nakanishi T., Hashimoto M., Mori Y., Osaka T. (2014). Field Effect Transistor Biosensor Using Antigen Binding Fragment for Detecting Tumor Marker in Human Serum. Materials.

[26] Lin C.-H., Chu C.-J., Teng K.-N., Su Y.-J., Chen C.-D., Tsai L.-C., Yang Y.-S. (2012). Recovery Based Nanowire Field-Effect Transistor Detection of Pathogenic Avian Influenza DNA. Jpn. J. Appl. Phys..

[27] Chen W.-Y., Chen H.-C., Yang Y.-S., Huang C.-J., Chan H.W.-H., Hu W.-P. (2013). Improved DNA Detection by Utilizing Electrically Neutral DNA Probe in Field-Effect Transistor Measurements as Evidenced by Surface Plasmon Resonance Imaging. Biosens. Bioelectron..

[28] Hu W.-P., Tsai C.-C., Yang Y.-S., Chan H.W.-H., Chen W.-Y. (2018). Synergetic Improvements of Sensitivity and Specificity of Nanowire Field Effect Transistor Gene Chip by Designing Neutralized DNA as Probe. Sci. Rep..

[29] Patolsky F., Zheng G., Hayden O., Lakadamyaly M., Zhuang X., Lieber C.M. (2004). Electrical Detection of Single Viruses. Proc. Natl. Acad. Sci. USA.

[30] Zhang G.-J., Zhang L., Huang M.J., Luo Z.H.H., Tay G.K.I., Lim E.-J.A., Kang T.G., Chen Y. (2010). Silicon Nanowire Biosensor for Highly Sensitive and Rapid Detection of Dengue Virus. Sens. Actuators B Chem..

[31] Lin C.-H., Hsiao C.-Y., Hung C.-H., Lo Y.-R., Lee C.-C., Su C.-J., Lin H.-C., Ko F.-H., Huang T.-Y., Yang Y.-S. (2008). Ultrasensitive Detection of Dopamine Using a Polysilicon Nanowire Field-Effect Transistor. Chem. Commun..

[32] Wu C.-C., Ko F.-H., Yang Y.-S., Hsia D.-L., Lee B.-S., Su T.-S. (2009). Label-Free Biosensing of a Gene Mutation Using a Silicon Nanowire Field-Effect Transistor. Biosens. Bioelectron..

[33] Lin M.-Y., Hsu W.-Y., Yang Y.-S., Huang J.-W., Chung Y.-L., Chen H. (2016). Immobilized Rolling Circle Amplification on Extended-Gate Field-Effect Transistors with Integrated Readout Circuits for Early Detection of Platelet-Derived Growth Factor. Anal. Bioanal. Chem..

[34] Lo Y.-R., Chen H.-M.P., Yang Y.-S., Lu M.-P. (2018). Gas Sensing Ability on Polycrystalline-Silicon Nanowire. ECS J. Solid State Sci. Technol..

[35] Gao X.P.A., Zheng G., Lieber C.M. (2010). Subthreshold Regime Has the Optimal Sensitivity for Nanowire FET Biosensors. Nano Lett..

[36] Zheng G., Gao X.P.A., Lieber C.M. (2010). Frequency Domain Detection of Biomolecules Using Silicon Nanowire Biosensors. Nano Lett..

[37] Chu C.-J., Yeh C.-S., Liao C.-K., Tsai L.-C., Huang C.-M., Lin H.-Y., Shyue J.-J., Chen Y.-T., Chen C.-D. (2013). Improving Nanowire Sensing Capability by Electrical Field Alignment of Surface Probing Molecules. Nano Lett..

[38] Xie P., Xiong Q., Fang Y., Qing Q., Lieber C.M. (2012). Local Electrical Potential Detection of DNA by Nanowire–Nanopore Sensors. Nat. Nanotechnol..

[39] Gong J.-R. (2010). Label-Free Attomolar Detection of Proteins Using Integrated Nanoelectronic and Electrokinetic Devices. Small.

[40] Jiang X., Tian B., Xiang J., Qian F., Zheng G., Wang H., Mai L., Lieber C.M. (2011). Rational Growth of Branched Nanowire Heterostructures with Synthetically Encoded Properties and Function. Proc. Natl. Acad. Sci. USA.

[41] Stern E., Vacic A., Rajan N.K., Criscione J.M., Park J., Ilic B.R., Mooney D.J., Reed M.A., Fahmy T.M. (2010). Label-Free Biomarker Detection from Whole Blood. Nat. Nanotechnol..

[42] Ajayan P.M. (1999). Nanotubes from Carbon. Chem. Rev..

[43] Heller I., Kong J., Heering H.A., Williams K.A., Lemay S.G., Dekker C. (2005). Individual Single-Walled Carbon Nanotubes as Nanoelectrodes for Electrochemistry. Nano Lett..

[44] Krapf D., Quinn B.M., Wu M.-Y., Zandbergen H.W., Dekker C., Lemay S.G. (2006). Experimental Observation of Nonlinear Ionic Transport at the Nanometer Scale. Nano Lett..

[45] Gooding J.J., Chou A., Liu J., Losic D., Shapter J.G., Hibbert D.B. (2007). The Effects of the Lengths and Orientations of Single-Walled Carbon Nanotubes on the Electrochemistry of Nanotube-Modified Electrodes. Electrochem. Commun..

[46] Heller I., Janssens A.M., Mannik J., Minot E.D., Lemay S.G., Dekker C. (2008). Identifying the Mechanism of Biosensing with Carbon Nanotube Transistors. Nano Lett..

[47] Davis J.J., Coleman K.S., Azamian B.R., Bagshaw C.B., Green M.L.H. (2003). Chemical and Biochemical Sensing with Modified Single Walled Carbon Nanotubes. Chem. Eur. J..

[48] Gooding J.J., Wibowo R., Liu J., Yang W., Losic D., Orbons S., Mearns F.J., Shapter J.G., Hibbert D.B. (2003). Protein Electrochemistry Using Aligned Carbon Nanotube Arrays. J. Am. Chem. Soc..

[49] Li J., Ng H.T., Cassell A., Fan W., Chen H., Ye Q., Koehne J., Han J., Meyyappan M. (2003). Carbon Nanotube Nanoelectrode Array for Ultrasensitive DNA Detection. Nano Lett..

[50] Tsang S.C., Davis J.J., Green M.L.H., Hill H.A.O., Leung Y.C., Sadler P.J. (1995). Immobilization of Small Proteins in Carbon Nanotubes: High-Resolution Transmission Electron Microscopy Study and Catalytic Activity. J. Chem. Soc. Chem. Commun..

[51] Davis J.J., Green M.L.H., Hill H.A.O., Leung Y.C., Sadler P.J., Sloan J., Xavier A.V., Tsang S.C. (1998). The Immobilisation of Proteins in Carbon Nanotubes. Inorg. Chim. Acta.

[52] Liu Y., Wang M., Zhao F., Xu Z., Dong S. (2005). The Direct Electron Transfer of Glucose Oxidase and Glucose Biosensor Based on Carbon Nanotubes/Chitosan Matrix. Biosens. Bioelectron..

[53] Star A., Gabriel J.-C.P., Bradley K., Gruner G. (2003). Electronic Detection of Specific Protein Binding Using Nanotube FET Devices. Nano Lett..

[54] Star A., Tu E., Niemann J., Gabriel J.-C.P., Joiner C.S., Valcke C. (2006). Label-Free Detection of DNA Hybridization Using Carbon Nanotube Network Field-Effect Transistors. Proc. Natl. Acad. Sci. USA.

[55] Zhang Y., Zheng L. (2010). Towards Chirality-Pure Carbon Nanotubes. Nanoscale.

[56] Li Z., Liu Z., Sun H., Gao C. (2015). Superstructured Assembly of Nanocarbons: Fullerenes, Nanotubes, and Graphene. Chem. Rev..

[57] Lee C., Wei X., Kysar J.W., Hone J. (2008). Measurement of the Elastic Properties and Intrinsic Strength of Monolayer Graphene. Science.

[58] Chandran G.T., Li X., Ogata A., Penner R.M. (2017). Electrically Transduced Sensors Based on Nanomaterials (2012–2016). Anal. Chem..

[59] Bolotin K.I., Sikes K.J., Jiang Z., Klima M., Fudenberg G., Hone J., Kim P., Stormer H.L. (2008). Ultrahigh Electron Mobility in Suspended Graphene. Solid State Commun..

[60] Georgakilas V., Perman J.A., Tucek J., Zboril R. (2015). Broad Family of Carbon Nanoallotropes: Classification, Chemistry, and Applications of Fullerenes, Carbon Dots, Nanotubes, Graphene, Nanodiamonds, and Combined Superstructures. Chem. Rev..

[61] Chen S., Liu L., Zhou J., Jiang S. (2003). Controlling Antibody Orientation on Charged Self-Assembled Monolayers. Langmuir.

[62] Chou W.-C., Hu W.-P., Yang Y.-S., Chan H.W.-H., Chen W.Y. (2019). Neutralized Chimeric DNA Probe for the Improvement of GC-Rich RNA Detection Specificity on the Nanowire Field-Effect Transistor. Sci. Rep..

[63] Xu S., Zhang C., Jiang S., Hu G., Li X., Zou Y., Liu H., Li J., Li Z., Wang X. (2019). Graphene Foam Field-Effect Transistor for Ultra-Sensitive Label-Free Detection of ATP. Sens. Actuators B Chem..

[64] Thakur B., Zhou G., Chang J., Pu H., Jin B., Sui X., Yuan X., Yang C.-H., Magruder M., Chen J. (2018). Rapid Detection of Single *E. coli* Bacteria Using a Graphene-Based Field-Effect Transistor Device. Biosens. Bioelectron..

[65] Ren R., Zhang Y., Nadappuram B.P., Akpinar B., Klenerman D., Ivanov A.P., Edel J.B., Korchev Y. (2017). Nanopore Extended Field-Effect Transistor for Selective Single-Molecule Biosensing. Nat. Commun..

[66] Shariati M. (2018). The Field Effect Transistor DNA Biosensor Based on ITO Nanowires in Label-Free Hepatitis B Virus Detecting Compatible with CMOS Technology. Biosens. Bioelectron..

[67] Bhat K.S., Ahmad R., Yoo J.-Y., Hahn Y.-B. (2017). Nozzle-Jet Printed Flexible Field-Effect Transistor Biosensor for High Performance Glucose Detection. J. Colloid Interface Sci..

[68] Fathollahzadeh M., Hosseini M., Norouzi M., Ebrahimi A., Fathipour M., Kolahdouz M., Haghighi B. (2018). Immobilization of Glucose Oxidase on ZnO Nanorods Decorated Electrolyte-Gated Field Effect Transistor for Glucose Detection. J. Solid State Electrochem..

[69] Ahmad R., Ahn M.-S., Hahn Y.-B. (2017). ZnO Nanorods Array Based Field-Effect Transistor Biosensor for Phosphate Detection. J. Colloid Interface Sci..

[70] Bao Z., Sun J., Zhao X., Li Z., Cui S., Meng Q., Zhang Y., Wang T., Jiang Y. (2017). Top-Down Nanofabrication of Silicon Nanoribbon Field Effect Transistor (Si-NR FET) for Carcinoembryonic Antigen Detection. Int. J. Nanomed..

[71] Ma S., Li X., Lee Y.-K., Zhang A. (2018). Direct Label-Free Protein Detection in High Ionic Strength Solution and Human Plasma Using Dual-Gate Nanoribbon-Based Ion-Sensitive Field-Effect Transistor Biosensor. Biosens. Bioelectron..

[72] Ma S., Lee Y.-K., Zhang A., Li X. (2018). Label-Free Detection of Cordyceps Sinensis Using Dual-Gate Nanoribbon-Based Ion-Sensitive Field-Effect Transistor Biosensor. Sens. Actuators B Chem..

[73] Nguyen T.T.T., Legallais M., Morisot F., Cazimajou T., Mouis M., Salem B., Stambouli B., Ternon C. (2017). On the Development of Label-Free DNA Sensor Using Silicon Nanonet Field-Effect Transistors. Proceedings.

[74] Chu C.-H., Sarangadharan I., Regmi A., Chen Y.-W., Hsu C.-P., Chang W.-H., Lee G.-Y., Chyi J.-I., Chen C.-C., Shiesh S.-C. (2017). Beyond the Debye Length in High Ionic Strength Solution: Direct Protein Detection with Field-Effect Transistors (FETs) in Human Serum. Sci. Rep..

[75] Chen P.-C., Chen Y.-W., Sarangadharan I., Hsu C.-P., Chen C.-C., Shiesh S.-C., Lee G.-B., Wang Y.-L. (2017). Editors’ Choice—Field-Effect Transistor-Based Biosensors and a Portable Device for Personal Healthcare. ECS J. Solid State Sci. Technol..

[76] Kao W.-C., Chen Y.-W., Chu C.-H., Chang W.-H., Shiesh S.-C., Wang Y.-L., Lee G.-B. (2017). Detection of C-reactive Protein on an Integrated Microfluidic System by Utilizing Field-Effect Transistors and Aptamers. Biomicrofluidics.

[77] Sinha A., Tai T.-Y., Lee G.-B., Wang Y.-L. (2018). Integrated Microfluidic System with Field Effect Transistor for Automatic Detection of Multiple Cardiovascular Biomarkers. IEEE Micro Electro Mechanical Systems.

[78] Stock D., Muntze G.M., Figge S., Eickhoff M. (2018). Ion Sensitive AlGaN/GaN Field-Effect Transistors with Monolithically Integrated Wheatstone Bridge for Temperature- and Drift Compensation in Enzymatic Biosensors. Sens. Actuators B Chem..

[79] Jang H.-J., Lee T., Song J., Russell L., Li H., Dailey J., Searson P.C., Katz H.E. (2018). Electronic Cortisol Detection Using an Antibody-Embedded Polymer Coupled to a Field-Effect Transistor. ACS Appl. Mater. Interfaces.

[80] Jeong G., Oh J., Jang J. (2019). Fabrication of N-Doped Multidimensional Carbon Nanofibers for High-Performance Cortisol Biosensors. Biosens. Bioelectron..

[81] Chen Y., Ren R., Pu H., Chang J., Mao S., Chen J. (2017). Field-Effect Transistor Biosensors with Two-Dimensional Black Phosphorus Nanosheets. Biosens. Bioelectron..

[82] Seshadri P., Manoli K., Schneiderhan-Marra N., Anthes U., Wierzchowiec P., Bonrad K., Franco C.D., Torsi L. (2018). Low-Picomolar, Label-Free Procalcitonin Analytical Detection with an Electrolyte-Gated Organic Field-Effect Transistor Based Electronic Immunosensor. Biosens. Bioelectron..

[83] Shi W., Yu J., Katz H.E. (2018). Sensitive and Selective Pentacene-Guanine Field-Effect Transistor Sensing of Nitrogen Dioxide and Interferent Vapor Analytes. Sens. Actuators B Chem..

[84] Chen Y., Ren R., Pu H., Guo X., Chang J., Zhou G., Mao S., Kron M., Chen J. (2017). Field-Effect Transistor Biosensor for Rapid Detection of Ebola Antigen. Sci. Rep..

[85] Lin Y.-H., Lin W.-S., Wong J.-C., Hsu W.-C., Peng Y.-S., Chen C.-L. (2017). Bottom-Up Assembly of Silicon Nanowire Conductometric Sensors for the Detection of Apolipoprotein A1, a Biomarker for Bladder Cancer. Microchim. Acta.

[86] Presnova G., Presnov D., Krupenin V., Grigorenko V., Trifonov A., Andreeva I., Ignatenko O., Egorov A., Rubtsova M. (2017). Biosensor Based on a Silicon Nanowire Field-Effect Transistor Functionalized by Gold Nanoparticles for the Highly Sensitive Determination of Prostate Specific Antigen. Biosens. Bioelectron..

[87] Rubtsova M., Presnova G., Presnov D., Krupenin V., Grigorenko V., Egorov A. (2017). Biosensor Based on a Nanowire Field-Effect Transistor for the Determination of Prostate Specific Antigen. Procedia Technol..

[88] Lei Y.-M., Xiao M.-M., Li Y.-T., Xu L., Zhang H., Zhang Z.-Y., Zhang G.-J. (2017). Detection of Heart Failure-Related Biomarker in Whole Blood with Graphene Field Effect Transistor Biosensor. Biosens. Bioelectron..

[89] Munief W.-M., Lu X., Teucke T., Wilhelm J., Britz A., Hempel F., Lanche R., Schwartz M., Law J.K.Y., Grandthyll S. (2019). Reduced Graphene Oxide Biosensor Platform for the Detection of NT-ProBNP Biomarker in Its Clinical Range. Biosens. Bioelectron..

[90] Islam S., Shukla S., Bajpaj V.K., Han Y.-K., Huh Y.S., Kumar A., Ghosh A., Gandhi S. (2019). A Smart Nanosensor for the Detection of Human Immunodeficiency Virus and Associated Cardiovascular and Arthritis Diseases Using Functionalized Graphene-Based Transistors. Biosens. Bioelectron..

[91] Si K., Cheng S., Hideshima S., Kuroiwa S., Nakanishi T., Osaka T. (2018). Multianalyte Detection of Cancer Biomarkers in Human Serum Using Label-Free Field Effect Transistor Biosensor. Sens. Mater..

[92] Islam S., Shukla S., Bajpaj V.K., Han Y.-K., Huh Y.S., Ghosh A., Gandhi S. (2019). Microfluidic-Based Graphene Field Effect Transistor for Femtomolar Detection of Chlorpyrifos. Sci. Rep..

[93] Berto M., Vecchi E., Baiamonte L., Condo C., Sensi M., Lauro M.D., Sola M., Stradis A.D., Biscarini F., Minafra A. (2019). Label Free Detection of Plant Viruses with Organic Transistor Biosensors. Sens. Actuators B Chem..

[94] Napoli C., Lai S., Giannetti A., Tombelli S., Baldini F., Barbaro M., Bonfiglio A. (2018). Electronic Detection of DNA Hybridization by Coupling Organic Field-Effect Transistor-Based Sensors and Hairpin-Shaped Probes. Sensors.

[95] Gao Z., Xia H., Zauberman J., Tomaiuolo M., Ping J., Zhang Q., Ducos P., Ye H., Wang S., Yang X. (2018). Detection of Sub-fM DNA with Target Recycling and Self-Assembly Amplification on Graphene Field-Effect Biosensors. Nano Lett..

[96] Mei J., Li Y.-T., Zhang H., Xiao M.-M., Ning Y., Zhang Z.-Y., Zhang G.-J. (2018). Molybdenum Disulfide Field-Effect Transistor Biosensor for Ultrasensitive Detection of DNA by Employing Morpholino as Probe. Biosens. Bioelectron..

[97] Liu J., Chen X., Wang Q., Xiao M., Zhong D., Sun W., Zhang G., Zhang Z. (2019). Ultrasensitive Monolayer MoS_2_ Field-Effect Transistor Based DNA Sensors for Screening of Down Syndrome. Nano Lett..

[98] Campos R., Borme J., Guerreiro J.R., Machado G., Cerqueira M.F., Petrovykh D.Y., Alpuim P. (2019). Attomolar Label-Free Detection of DNA Hybridization with Electrolyte-Gated Graphene Field-Effect Transistors. ACS Sens..

[99] Fatin M.F., Ruslinda A.R., Gopinath S.C.B., Arshad M.K.M. (2019). High-Performance Interactive Analysis of Split Aptamer and HIV-1 Tat on Multiwall Carbon Nanotube-Modified Field-Effect Transistor. Int. J. Biol. Macromol..

[100] Majd S.M., Salimi A., Astinchap B. (2018). The Development of Radio Frequency Magnetron Sputtered P-Type Nickel Oxide Thin Film Field-Effect Transistor Device Combined with Nucleic Acid Probe for Ultrasensitive Label-Free HIV-1 Gene Detection. Sens. Actuators B Chem..

[101] Singh N.K., Thungon P.D., Estrela P., Goswami P. (2019). Development of an Aptamer-Based Field Effect Transistor Biosensor for Quantitative Detection of Plasmodium Falciparum Glutamate Dehydrogenase in Serum Samples. Biosens. Bioelectron..

[102] Hao Z., Zhu Y., Wang X., Rotti P.G., DiMarco C., Tyler S.R., Zhao X., Engelhardt J.F., Hone J., Lin Q. (2017). Real-Time Monitoring of Insulin Using a Graphene Field-Effect Transistor Aptameric Nanosensor. ACS Appl. Mater. Interfaces.

[103] Nakatsuka N., Yang K.-A., Abendroth J.M., Cheung K.M., Xu X., Yang H., Zhao C., Zhu B., Rim Y.S., Yang Y. (2018). Aptamer–Field-Effect Transistors Overcome Debye Length Limitations for Small-Molecule Sensing. Science.

[104] Hideshima S., Wustoni S., Kobayashi M., Hayashi H., Kuroiwa S., Nakanishi T., Osaka T. (2018). Effect of Human Serum on the Electrical Detection of Amyloid-β Fibrils in Biological Environments Using Azo-Dye Immobilized Field Effect Transistor (FET) Biosensor. Sens. Biosens. Res..

[105] Gutierrez-Sanz O., Andoy N.M., Filipiak M.S., Haustein N., Tarasov A. (2017). Direct, Label-Free, and Rapid Transistor-Based Immunodetection in Whole Serum. ACS Sens..

[106] Andoy N.M., Filipiak M.S., Vettel D., Gutierrez-Sanz O., Tarasov A. (2018). Graphene-Based Electronic Immunosensor with Femtomolar Detection Limit in Whole Serum. Adv. Mater. Technol..

[107] Filipiak M.S., Rother M., Andoy N.M., Knudsen A.C., Grimm S., Bachran C., Swee L.K., Zaumseil J., Tarasov A. (2018). Highly Sensitive, Selective and Label-Free Protein Detection in Physiological Solutions Using Carbon Nanotube Transistors with Nanobody Receptors. Sens. Actuators B Chem..

[108] Hideshima S., Saito M., Fujita K., Harada Y., Tsuna M., Sekiguchi S., Kuroiwa S., Nakanishi T., Osaka T. (2018). Label-free Detection of Allergens in Food via Surfactant-Induced Signal Amplification Using a Field Effect Transistor-Based Biosensor. Sens. Actuators B Chem..

[109] Gao N., Zhou W., Jiang X., Hong G., Fu T.-M., Lieber C.-M. (2015). General Strategy for Biodetection in High Ionic Strength Solutions Using Transistor-Based Nanoelectronic Sensors. Nano Lett..

[110] Haustein N., Gutierrez-Sanz O., Tarasov A. (2019). Analytical Model to Describe the Effect of Polyethylene Glycol on Ionic Screening of Analyte Charges in Transistor-Based Immusisssnosensing. ACS Sens..

